# The Infant Skin Barrier: Can We Preserve, Protect, and Enhance the Barrier?

**DOI:** 10.1155/2012/198789

**Published:** 2012-09-04

**Authors:** Lorena S. Telofski, A. Peter Morello, M. Catherine Mack Correa, Georgios N. Stamatas

**Affiliations:** ^1^JOHNSON & JOHNSON Consumer Companies, Inc., 199 Grandview Road, Skillman, NJ 08558, USA; ^2^Evidence Scientific Solutions, 123 South Broad Street, Suite 1670, Philadelphia, PA 19109, USA; ^3^JOHNSON & JOHNSON Santé Beauté France, 1 rue Camille Desmoulins, 92787 Issy-les-Moulineaux, France

## Abstract

Infant skin is different from adult in structure, function, and composition. Despite these differences, the skin barrier is competent at birth in healthy, full-term neonates. The primary focus of this paper is on the developing skin barrier in healthy, full-term neonates and infants. Additionally, a brief discussion of the properties of the skin barrier in premature neonates and infants with abnormal skin conditions (i.e., atopic dermatitis and eczema) is included. As infant skin continues to mature through the first years of life, it is important that skin care products (e.g., cleansers and emollients) are formulated appropriately. Ideally, products that are used on infants should not interfere with skin surface pH or perturb the skin barrier. For cleansers, this can be achieved by choosing the right type of surfactant, by blending surfactants, or by blending hydrophobically-modified polymers (HMPs) with surfactants to increase product mildness. Similarly, choosing the right type of oil for emollients is important. Unlike some vegetable oils, mineral oil is more stable and is not subject to oxidation and hydrolysis. Although emollients can improve the skin barrier, more studies are needed to determine the potential long-term benefits of using emollients on healthy, full-term neonates and infants.

## 1. Introduction

Skin barrier function resides primarily within the stratum corneum (SC), the top layer of the epidermis. Although the SC is only 7–35 *μ*m thick [1, 2], it plays a vital role in forming a protective barrier and helps to prevent percutaneous entry of harmful pathogens into the body [3, 4]. In addition to serving as a physical barrier, the SC has other important functions, including engaging in thermoregulation, gas exchange, and maintenance of proper hydration. The SC also serves important functions in innate immunity [5] and its slightly acidic pH [6] provides additional protection against pathogens.

Maintenance of the skin barrier is essential for survival [1]. This is especially true for neonates and infants because their skin differs from mature adult skin in structure, function, and composition [1, 2, 7] and is particularly susceptible to infection [3]. During the late fetal period (20 weeks to birth), skin becomes functional and develops a protective barrier [8]. Although full-term infants are born with a competent skin barrier [9, 10], their skin is still developing through the first year of life [2, 11]. During the postnatal period, even the composition of commensal bacteria residing on the skin surface differs from that of adults and continues to evolve over the first year of life [12].

Given that skin continues to develop through the first year of life, the use of appropriate, evidence-based skin care practices is important. Maintaining skin barrier function is critical to preventing organ dehydration [13]. The SC water content is involved in maintaining SC structural integrity and functionality [14]. It is generally accepted that recommendations for infant skin care regimens should be evidence-based [15]. Although several studies have evaluated nonprescription emollient strategies to improve barrier function [16, 17] or improve fluid and electrolyte balance [18] in neonates, infants, or children with compromised skin, limited information is available on skin care regimens that enable maintenance or enhancement of skin barrier integrity in normal neonatal or infant skin [19, 20].

Skin cleansing and emollient use are two simple strategies that can help keep skin healthy. Proper skin cleansing helps keep infant skin free of unwanted irritants, including saliva, nasal secretions, urine, feces, fecal enzymes, dirt, and microbial pathogens. Exposure to such factors for long periods, especially in the diaper region, can lead to discomfort, irritation, infection, and skin barrier breakdown. In many cases, water alone is not sufficient to cleanse skin during bathing [21]. Epidemiologic studies and anecdotal reports have even suggested a possible link between household use of hard water and atopic eczema in children [22, 23], though a causal relationship has not been shown [24, 25].

In addition to using cleansers during bathing, emollient use during or after bathing also may have benefits [16–20, 26–29]. Emollients decrease transepidermal water loss (TEWL) [16, 17, 26], improve skin condition [17, 26], and may even lead to reduced mortality in extremely premature infants [28]. In adults, 7 weeks of emollient use led to improvement in skin barrier function [27].

In this paper, we discuss the unique structure, function, and composition of infant skin, the importance of maintaining skin barrier integrity, and best practices for maintaining or improving infant epidermal barrier function, including use of appropriately formulated cleansers and emollients. We also discuss various neonatal and infant skin care guidelines from around the world and some controversies surrounding these guidelines. Finally, we will explore the idea that the onset of emollient use from birth may play a role in preserving and protecting the infant skin barrier later in life.

## 2. Infant Skin: Structure, Function, and Composition

Infant skin is different from adult skin: it undergoes a maturation process through at least the first year of life [2, 7, 11]. Several groups have measured or compared the epidermis of infants and adults [1, 2, 9, 30]. In one study, the epidermis of full-term neonates at birth was found to have 4.3 ± 0.7 cell layers that were vertically stacked from the basal layer to the stratum granulosum (excluding the SC), whereas the epidermis of preterm neonates at birth had only 2.9 ± 0.5 cell layers [9]. In their review of the literature, Chiou and Blume-Peytavi [1] reported that SC thickness ranged from 5.6 *μ*m to 35.4 *μ*m for infants and 15.2 *μ*m to 35.4 *μ*m for adults. Our group found that the suprapapillary epidermis and the SC had respective thicknesses that were on average 20% and 30% thinner in infants than in adults [2]. On the lower thigh area, infant SC was determined to be 7.3 ± 1.1 *μ*m, whereas adult SC on the same region was 10.5 ± 2.1 *μ*m [2].

At birth, full-term neonates have competent barrier function [10, 13] and an epidermis that appears to be fully differentiated [9], but closer examination reveals subtle structural and morphologic differences between infant and adult skin [2]. These differences may lead to observable functional differences between infant and adult skin [11].  Table 1 contains an overview of the major similarities and differences between infant and adult skin.

**Figure 1:**
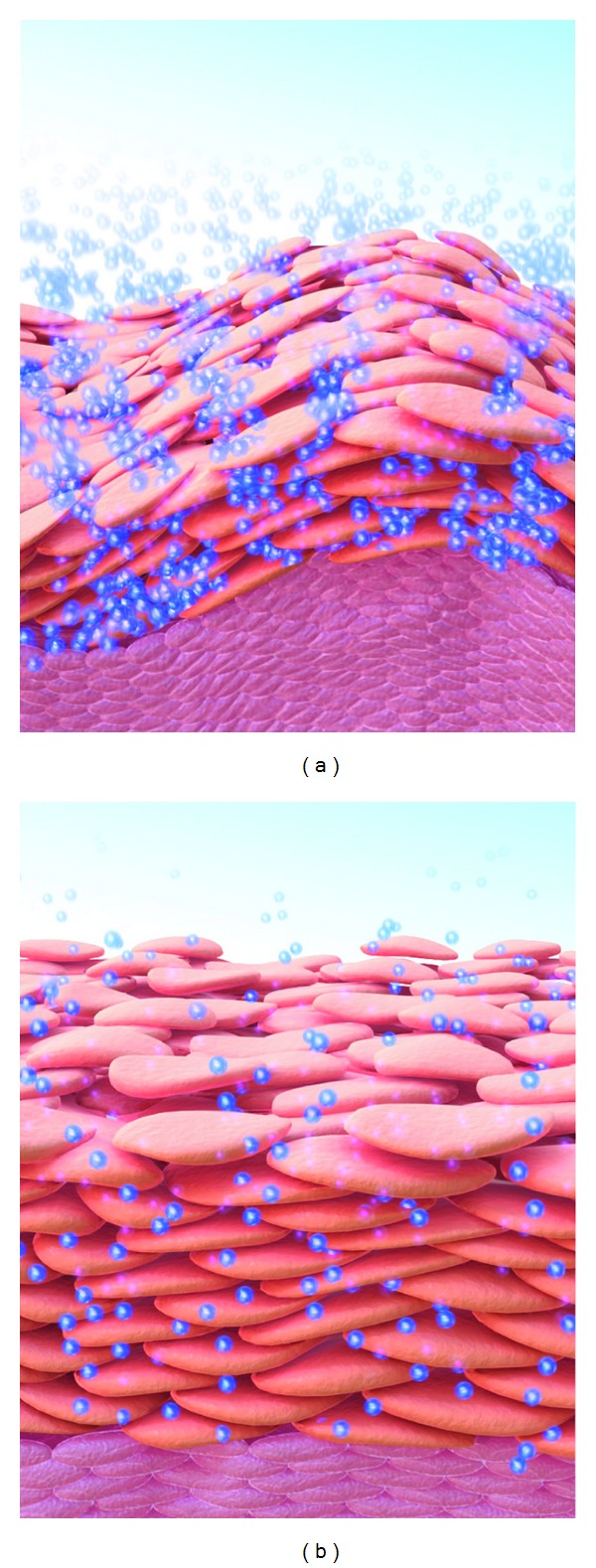
Infant and adult skin: stratum corneum (SC) hydration and water transport properties. The SC of infant skin (a) and adult skin (b) is hydrated (small blue spheres) under normal conditions. Infant SC is more hydrated but also loses water at higher rates than adult SC [11].

The water-handling properties of infant skin are unique and distinct from adult skin.  Figure 1 shows a schematic of infant and adult SC hydration and their respective water-holding properties. Neonatal skin after birth is considerably drier compared with that of adults [31, 32]. However, during the first month of life, the difference in SC hydration between infants and adults is reversed [32, 33], leading to increased skin hydration in older infants (aged 3–24 months) relative to adult skin [11, 34]. As skin becomes more hydrated, the SC that is initially rough smoothens [32].

In addition to undergoing structural and functional changes, the composition of the cutaneous microflora evolves over the first year of life [12]. Although adult skin is colonized mostly by the phyla Proteobacteria, Actinobacteria, and Firmicutes, the order of predominance changes in infant skin to Firmicutes (predominantly *Staphylococci*), followed by Actinobacteria, Proteobacteria, and Bacteroidetes [12]. Although the implications of these findings are not yet known, early microbial colonization is expected to influence the development of immune function in skin. It also will be important to characterize the further evolution of the human skin microbiome during the first few years of life to determine if commensal bacteria play a role in the maintenance of skin barrier function beyond serving as sentinels of innate immune defense [35].

## 3. The Skin Barrier Is Competent at Birth in Healthy, Full-Term Neonates

After birth, skin barrier function is influenced by the shift from an aqueous, warm environment in utero to a cooler, arid, and more variable extrauterine world [11, 36]. Skin development is contingent on gestational age. As gestation increases, the thickness and the number of cell layers in the epidermis increase [9]. Morphologic changes also occur, including the formation of an increasingly undulated dermoepidermal junction [9]. Histologically, a well-developed epidermis emerges at 34 weeks of gestation [9], though the period required for complete SC maturation has been reported to vary between 30 and 37 weeks [10].

Although infant skin is different from adult skin [2, 11], studies assessing the histologic and biophysical properties of the SC have demonstrated that the skin barrier is competent at birth in healthy, full-term neonates to prevent organ dehydration [9, 10, 13]. The barrier properties of the skin depend greatly on the thickness and integrity of the SC [8, 9]. As would be expected, preterm infants have a skin barrier that is underdeveloped compared with full-term neonates [9]. In one study [9], the epidermal thickness of full-term neonates at birth was 43 ± 7 *μ*m versus 31 ± 7 *μ*m for preterm infants (24–30 weeks of gestation). 

In addition to SC thickness, other parameters can be used to assess barrier function, including skin water-handling properties [11, 34]. Water barrier function and skin hydration status are interdependent factors, the former of which is influenced largely by the organization and composition of the intercellular lipid matrix [37], natural moisturizing factor [38], and the permeation path length through the SC [39]. Skin water content also influences skin barrier function by regulating the activity of hydrolytic enzymes that are involved in SC maturation and corneocyte desquamation [40].

Researchers can assess the skin's capacity to absorb and retain water with sorption-desorption tests that use electrical measurements (e.g., skin surface conductance or capacitance) before and after topical application of water on the skin surface [1, 41]. Water barrier function, which affects rates of water absorption and desorption, is localized within the SC [42] and has been shown to vary between infants and adults [11, 31]. In addition, water content within the SC can have a profound effect on skin surface morphology [43], desquamation [44], and epidermal expression of keratins and cornified envelope proteins [45].

Newborn skin has been reported to have lower skin surface hydration and increased water loss compared with skin from 1- to 6-month-old infants or adults [31]. Our group also found that infant skin (3–12 months) on the upper ventral arm and lower dorsal arm gained and lost water at significantly faster rates than the same regions on adult skin [11]. Skin surface hydration on the upper ventral arm and lower dorsal arm was greater in infants than adults. The distribution of water in the SC varied between infants and adults based on water concentration profiles calculated using confocal Raman microspectroscopy. Infants had more water on the skin surface, more water within the SC, and more water distributed throughout the first 26 *μ*m below the skin surface. Infant SC also had a steeper water gradient compared with adult skin.

TEWL is a noninvasive method that can be used to monitor changes in SC barrier function [46]; it also enables dynamic measurement of water loss [11]. High basal TEWL is suggestive of incomplete skin barrier function and is indirectly proportional to the integrity of water barrier function. This method has been used to confirm that epidermal permeability barrier function is developed fully at birth in full-term neonates [6, 10]. In older infants (3–12 months) our group found that TEWL was significantly higher compared with adult skin (*P *< .0005; 3–6 and 7–12 months old versus adult) [11]. 

Formation of an acidic SC is essential for epidermal barrier maturation and repair processes [10]. Many factors contribute to formation of the acid mantle, including sebum secretion, sweat (lactic acid), amino acids and amino acid derivatives (urocanic acid and pyrrolidone carboxylic acid), and exocytosis of lamellar body contents at the stratum granulosum/stratum compactum interface [47]. At birth, full-term neonates have a skin surface pH that varies between 6.34 and 7.5 [6, 48]. Within the first 2 weeks of life, skin surface pH falls to approximately 5 [3, 48], which is similar to the skin surface pH that has been observed during adulthood (pH range: 4.0 to 6.7) [6, 49]. Discrepancies in skin surface pH between studies could be the result of differences in participant age (infant versus child), gender mismatch, body location (volar forearm versus buttock), or instrumentation. It should be noted that adult skin surface pH also has been shown to vary by a wide margin [49]. Taken together, published data indicate that skin surface pH is close to neutral at birth and becomes more acidic over the first few days of life. Within a matter of weeks, skin surface pH is similar to levels observed in adults. However, consensus has not been reached on the duration of this transition period.

## 4. Maintenance of Skin Barrier Integrity Is Essential to Overall Health and Wellness

Skin barrier function is essential for survival [1] and is critical to preventing percutaneous entry of bacteria and other pathogens into neonatal skin [50]. If the skin barrier is disturbed, bacteria or bacterial factors will have access to living epidermal keratinocytes and can induce defensive immune responses [4]. Keratinocytes produce antimicrobial peptides (AMPs), including the cathelicidin-derived peptide LL-37 and human *β*-defensins 1-3 [4]. In the absence of AMPs, pathogenic microorganisms can invade the surface of skin, leading to infection or an imbalance of commensal flora versus pathogenic bacteria. For example, patients suffering from burns, chronic wounds, surgery, or injuries that are associated with skin barrier dysfunction are more susceptible to infections caused by *Pseudomonas aeruginosa* [4], yet this opportunistic pathogen rarely causes infections on healthy human skin [4].

## 5. Abnormal Infant Skin Conditions and Barrier Integrity

### 5.1. Atopic Dermatitis (AD)

During childhood, skin disorders that are characterized by skin barrier dysfunction are common. Compromised skin barrier integrity is thought to be critical to the early onset and severity of AD, which is often accompanied by dry, scaly skin. AD is an inflammatory skin condition that occurs in 15–20% of children [51, 52]. Alterations in skin barrier properties that are observed in AD include increased TEWL [53], changes in skin surface pH [54], increased skin permeability [55], increased bacterial colonization [56], alterations in AMP expression [57], and compromised skin permeability barrier integrity [58]. Once the skin barrier is compromised, allergens, irritants, and other unwanted agents can penetrate skin, leading to aggravation of symptoms associated with AD.

There are several guidelines that discuss how caregivers can manage and treat AD [59, 60]. Recommendations to relieve AD include using warm water in lieu of hot water, taking short baths (5–10 minutes), and using a liquid cleanser with emollient that does not compromise skin barrier integrity, followed by gentle dry patting with a soft towel and immediate application of a skin emollient [29, 61]. 

The Royal College of Paediatrics and Child Health (RCPCH) presented a tiered approach to the management of mild, moderate, and severe atopic eczema [62]. In all three cases, the RCPCH noted that initial treatment should focus on repairing the skin barrier through the use of emollients for moisturizing, washing, and bathing. Depending on severity, emollient use can be supplemented with topical corticosteroids. In cases of moderate atopic eczema, bandages and topical calcineurin inhibitors (second-line treatment) can be used to supplement emollient use. During severe atopic eczema, emollient use can be supplemented with phototherapy and systemic therapy. 

### 5.2. Irritant Diaper Dermatitis

Irritant diaper dermatitis is a complex skin condition that is characterized by compromised epidermal barrier function occurring on the buttocks, perianal region, inner thighs, and abdomen. Skin occlusion, friction, lipolytic and proteolytic activity of fecal enzymes, increased skin surface pH, and prolonged exposure to urine are all contributing factors to the onset of irritant diaper dermatitis [63]. Greater than 50% of infants will have at least one episode of irritant diaper dermatitis during the diaper-wearing phase [64]. Clinical presentation of irritant diaper dermatitis includes skin erythema [65], but severe cases may lead to presentation of papules and edema [66].

Within the past 10 years, there have been several reviews discussing the etiology and management of irritant diaper dermatitis [67–71]. Although use of appropriately formulated cleansers and emollients can help maintain the epidermal skin barrier in the diaper region, good hygiene and adequate protection are necessary to prevent skin barrier breakdown, rash, and infection. 

## 6. Cleansing Is Vital to Maintaining Good Health and Hygiene

### 6.1. Infant Skin Care Guidelines, Recommendations, and Review of the Literature

Keeping babies clean and good skin hygiene are essential to overall health. Cleansing helps keep skin free of unwanted substances, including irritants (saliva, nasal secretions, urine, feces, and fecal enzymes), dirt, and transient germs.Keeping hands clean, particularly in the case of babies with their hand-to-mouth behaviors, can help reduce or prevent oral transmission of microbial contaminants. Caregivers should give special attention to skin on the facial area, which may be irritated easily by milk, food, and saliva.Skin folds and creases on the face also should be kept clean.

Although the benefits of good hygiene are known, neonatal skin cleansing and the use of cleansers, soaps, or other topicals during the bathing process is controversial. For most of the 20th century there were no formal guidelines on neonatal skin cleansing. In 1974, the American Academy of Pediatrics recommended that caregivers cleanse neonatal skin after the infant's temperature stabilizes [72]. In 1978, Sweden and Great Britain proposed similar recommendations [73]. In 2007, the Second Edition of the Association of Women's Health, Obstetric, and Neonatal Nurses (AWHONN) Neonatal Skin Care Evidence-Based Clinical Practice Guideline recommended that caregivers select mild cleansing bars or liquid cleansers that have a neutral pH (5.5 to 7.0) that are preservative-free or contain preservatives that have a demonstrated safety/tolerance profile [74]. In contrast, the National Institute for Clinical Excellence (NICE) clinical guideline 37 on postnatal care states the following [75]: “Parents should be advised that cleansing agents should not be added to a baby's bath water nor should lotions or medicated wipes be used. The only cleansing agent suggested, where it is needed, is a mild non-perfumed soap.” Despite these recommendations, there is limited evidence to support the NICE position on infant cleansing [29]. Water is insufficient for removal of all oil-soluble skin surface impurities [76, 77] and has poor pH-buffering action [78]. Depending on bathing frequency and quality of water used, washing with water alone can have a drying effect on infant skin [29], which may lead to impairment of infant skin condition. Although soap is an effective skin cleanser, it can disrupt skin surface pH, alter skin lipids, and cause dryness and irritation [79–81], all of which may make soap less preferable.

On 13 February 2007, a group of clinical experts in pediatrics and dermatology formed the first European Round Table meeting on “Best Practice for Infant Cleansing.” The consensus panel recommended that caregivers use liquid, pH-neutral, or mildly acidic cleansers over traditional alkaline soaps on neonates and infants [29]. In addition, the consensus panel made the following recommendations:

- Liquid cleansers are preferable to water alone.
- Liquid cleansers cleanse and hydrate skin better than water alone.
- Liquid preparations, which often contain emollients, are preferable to cleansing bars.
- Liquid cleansers should contain adequate and appropriate preservatives.
- An “ideal cleanser” is one that does not cause irritation, alterations to skin surface pH, or eye stinging.
- Skin care products should be selected on the basis of evidence acquired in practical use conditions.

Although the consensus panel recommended using liquid cleansers and believed that liquid cleansers have some desirable properties, to our knowledge no peer-reviewed publications have summarized the results from randomized controlled trials comparing the tolerance or efficacy of liquid or rinse-off cleansers to traditional soaps or syndet bars. In an open-label, controlled, randomized study, Gfatter et al. compared the effects of washing infant skin with a liquid detergent (pH 5.5), compact detergent (pH 5.5), or alkaline soap (pH 9.5) with a control group washing with water alone after a single wash [79]. Their study was designed to assess the effect of skin care regimens on pH, fat content, and skin hydration. Although all cleansing regimens tested (including the control) were shown to influence the parameters studied, the soap bar had the largest influence on skin pH and fat content, resulting in statistically higher pH (more alkaline) and statistically greater loss of fat. The study by Gfatter et al. concluded that the short-term effects from a single wash can disturb the skin acid mantle and its protective function, which suggests the need to determine the long-term effects of cleansing products and other skin care regimens [81].

Given the lack of harmonization across infant skin cleansing guidelines, bathing practices vary widely. Siegfried and Shah surveyed skin care cleansing practices in 15 neonatal nurseries from 12 hospitals in Missouri, Iowa, Illinois, and California [82]. Of these nurseries, four were defined as “low risk” and 11 were defined as “high risk.” Head nurses, nursery directors, or other healthcare professionals were asked questions about bathing practices, cord care, and general infant skin care. Bathing of full-term infants in the low-risk nurseries occurred on the first day when the infant was stable or when the infant's core temperature was 98.6°F. There was little variation in the cleansing products used during bathing. Nine of 15 nurseries used a mild baby cleanser. One nursery used more than one brand, and no information was given about the cleansing products used at the other five nurseries.

Garcia Bartels et al. evaluated the effect of bathing with or without a liquid cleanser on skin barrier function in healthy, full-term neonates [19]. TEWL, SC hydration, skin surface pH, and sebum were measured on the forehead, abdomen, upper leg, and buttock on day 2, week 2, 4, and 8 of life. After 8 weeks of life, skin surface pH was significantly lower in neonates who were bathed with a liquid cleanser versus those who were bathed with water alone. Bathing with a liquid cleanser did not lead to significant differences in median TEWL values or SC hydration on any of the tested body sites versus those who were bathed with water alone. Moreover, use of a liquid cleanser did not lead to statistically significant changes in sebum measurements. The use of a liquid cleanser was well tolerated in healthy, full-term neonates during the first 8 weeks of life. The study by Garcia Bartels et al. did not include premature neonates or infants with abnormal skin conditions and it is not known if similar observations would be made in premature neonates or those with compromised skin.

In a randomized, investigator-blinded clinical study, Dizon et al. compared the effects of twice-daily washing with water alone versus washing with water and a mild cleanser or water with a comparator cleanser for 2 weeks in 180 healthy infants [83]. After 2 weeks, cleansing with water alone led to a significant increase in erythema from baseline. In contrast, there was no change in skin erythema from baseline in the group that was cleansed with water and mild cleanser.

### 6.2. Formulation Considerations

Many traditional soaps contain detergents that are derived from saponification (e.g., the process of mixing a strongly alkaline solution with a fatty substance such as vegetable oil or tallow, leading to soap formation) [76]. Alkaline soaps can increase skin surface pH beyond what is considered an ideal range [76, 79]; they can also dissolve fat-soluble and water-soluble barrier components from the surface of skin [79]. Unlike traditional soaps, many of which can be irritating, infant cleansers should be mild to accommodate the maturing skin barrier. Infant cleansers should also wash away dirt, sebum, saliva, urine, fecal matter, and fecal enzymes with minimal effort [66, 80, 81]. 

Although most cleansers and soaps are suitable for adult bathing, cleansers for neonatal or infant skin should be formulated specifically for that population and its special needs. An ideal infant cleanser should contain at least one “surface-active agent” (surfactant), a molecule with both hydrophilic and oleophilic (lipophilic) properties that reduces the interfacial tension between oil and water. Surfactants enable formation of oil-in-water, water-in-oil, and more complex, multiphasic systems. By reducing interfacial tension, cleansers help to emulsify oils and other skin surface impurities into water [77], making their removal easier without requiring excessive friction or mechanical force during bathing.

Several classes of surfactants are used often in cleanser formulations, including anionic surfactants such as sodium lauryl sulfate (SLS) or sodium laureth sulfate (SLES), nonionic surfactants (e.g., poloxamers), and amphoteric surfactants (e.g., cocamidopropyl betaine). Foaming action and mildness are influenced by the charge of a surfactant's hydrophilic head group and the formation of spherical structures (micelles) that enable solubilization of oils and lipids from the skin surface [21]. Although anionic and amphoteric surfactants facilitate foam formation (a desirable aesthetic property for shampoo), they are usually less mild than nonionic surfactants such as polyethylene glycol (PEG)-80 sorbitan laurate.

Surfactant selection represents a tradeoff between functionality, aesthetics, and mildness. Due to their charge and ability to form smaller micelles relative to other surfactants, some anionic surfactants can be disruptive and irritating to skin [21, 81]. For example, SLS is an effective emulsifying and foaming agent, but in certain circumstances it may cause irritation [81, 84]. In contrast, PEGylated nonionic surfactants (e.g., PEG-80 sorbitan laurate or polyethylene oxides) can lead to micelle stabilization, potentially increasing cleanser mildness [21]. Cleansers containing sulfated ethoxylated alcohols (e.g., SLES), surfactants that have large head groups and have the ability to form larger micelles, may be formulated to have improved mildness compared with those containing SLS [84, 85]. In 20 healthy adult volunteers, patch testing revealed that SLES was milder and caused significantly less damage to the epidermal barrier compared with SLS [84]. After 7 days, no significant irritation was observed with SLES, even at the highest tested concentration (2.0%). Regeneration after skin irritation occurred much faster with SLES compared with similar concentrations of SLS [84]. In 2010, the Cosmetic Ingredient Review (CIR) panel concluded that SLES is safe as a cosmetic ingredient when used appropriately in products formulated to be nonirritating [86].

Mild moisturizing cleansers are expected to provide cleansing benefits without negatively altering the hydration and viscoelastic properties of skin [81]. Formulators can combine surfactants to create milder cleansers [21], which may be particularly ideal for individuals with AD [87]. For example, liquid body cleansers that contain a blend of anionic and amphoteric surfactants can be milder than a liquid cleanser that contains an equal proportion of anionic surfactant alone. The blending of hydrophobically-modified polymers (HMPs) with surfactants also may lead to increased cleanser mildness [88]. HMPs can interact with and associate with the hydrophobic tails of other surfactants, leading to self-assembly and the formation of larger surfactant/polymeric structures. The creation of micelles with a larger hydrodynamic diameter has been shown to have lower irritation potential and may ultimately allow for the creation of milder surfactant systems and better tolerated cleansers [88].

The properties of an ideal infant cleanser are summarized in Table 2. Traditional cleansers are formulated to have a pH that is similar to that of the skin surface. Liquid cleansers should be nonirritating and should enable maintenance of normal skin surface pH [29]. If the pH of a cleanser is acidic but does not perturb skin surface pH, it may be preferable to one that is pH neutral that causes a greater shift in skin surface pH. Solutions that are not pH neutral are not necessarily more irritating to skin. Moreover, it could be argued based on the weight of the evidence that alkaline cleansers would be least appropriate. Alkaline soap can disrupt skin surface pH [79], decrease SC thickness [89], decrease SC intracellular lipids [89], and lead to dryness and irritation [80, 81]. Buffer solutions with varying pH (4.0 to 10.5) were shown to be nonirritating to skin irrespective of pH [90]. In addition, detergents buffered at pH 3.5 or 7.0 caused similar levels of skin irritation [90]. Although cleansers can alter skin surface pH, temporary pH fluctuations may be stabilized by the skin's large buffering capacity [90]. A cleanser's effect on skin surface pH may be more important than the pH of the formulation itself in determining product mildness. 

There are conflicting reports in the literature about the effect of cleansers on cutaneous commensal bacteria. Maintaining a skin surface pH between 4.0 and 4.5 facilitates cutaneous commensal bacterial attachment to the surface of skin [49]. Larson and Dinulos hypothesized that inappropriately formulated soaps could alter the delicate balance between cutaneous commensal and pathogenic bacteria [3]. da Cunha and Procianoy investigated the effect of using a pH-neutral soap during bathing on cutaneous bacterial colonization in infants admitted to a neonatal intensive care unit [91]. After 1 week, the use of a pH-neutral soap did not have an effect on cutaneous bacterial colonization compared with infants who were bathed with water alone. Given the importance of cutaneous commensal bacteria to innate immunity [92], the use of mild cleansers that do not cause alterations in skin surface pH may be important for normal skin maturation and innate immune function.

### 6.3. Noninvasive Approaches to Predict Skin Irritation Potential

Interleukin-1*α* (IL-1*α*) and prostaglandin E_2_ mediate inflammation in skin via cytokine-dependent and arachidonic acid-dependent pathways, both of which play a role in the development of erythema and edema. Proinflammatory markers (including IL-1*α*) that are indicative of subclinical inflammation (i.e., erythema) may be useful in predicting the skin irritation potential of a skin cleansing product [93, 94].

Bernhofer et al. demonstrated that IL-1*α* can be a useful predictor of skin mildness and irritation potential [93]. Levels of subclinical irritation—even in the absence of visible erythema—can be determined using a noninvasive epidermal tape-stripping technique and enzyme-linked immunosorbent assay [95, 96]. IL-1 receptor antagonist (IL-1ra), IL-1*α*, and the ratio between these two molecules are useful for assessing skin reactivity [95] and measuring skin inflammation [95, 97]. The IL-1ra/IL-1*α* ratio increases during infancy, irritant diaper dermatitis, heat rash, and erythema [96]. By extension, the IL-1ra/IL-1*α* ratio also may help predict the irritation potential of skin cleansers [93]. It is anticipated that skin treated with a mild skin cleanser would have a lower IL-1ra/IL-1*α* ratio compared with skin treated with a more irritating cleanser, possibly leading to a more normalized skin condition.  Table 3 shows the proinflammatory activity of several commercially available cleansing products whose irritation potential was assessed by measuring IL-1*α* release using *in vitro* skin tissue equivalents (EpiDerm, MatTek Corporation, Ashland, MA, USA). A mild baby cleanser and mild baby shampoo caused less IL-1*α* release compared with a commercial sensitive skin syndet bar. Moreover, MTT cell proliferation (cell viability) assay data revealed that there was more cell cytotoxicity associated with the sensitive skin syndet bar. Although these data are from *in vitro* skin equivalents, the mild baby cleanser and mild baby shampoo would be expected to cause minimal release of IL-1*α* from infant skin, possibly leading to less skin irritation. Other methods for assessing cleanser mildness include measuring the percutaneous transit time, protein solubilization, or collagen-swelling potential [98].

## 7. Emollients Can Improve Skin Barrier Function in Healthy, Full-Term Neonates

Dry, scaly skin is common in neonates [31] but can occur at any stage of development. Although many factors contribute to skin surface hydration, the environment (i.e., dry, cold weather or wind) can accelerate the loss of moisture from the SC. Emollients have been used for centuries to protect the integrity of the SC and to maintain skin barrier function [99]. Appropriately formulated emollients can preserve, protect, and enhance the infant skin barrier by supplying the SC with water and lipids and by helping to inhibit water loss. Emollients also supply lipids to epidermal keratinocytes, where they can be transported through the cell membrane and metabolized within the cell [100]. Keratinocytes can then use lipids (including linoleic acid) as components to build a functional epidermal barrier [101].

Several studies have shown that emollient use can improve skin barrier function [16, 17] or improve fluid and electrolyte balance [18] in preterm infants, but very few studies have investigated the use of emollients on healthy, full-term neonates [19, 20]. Garcia Bartels et al. investigated the effect of applying topical emollients on healthy, full-term neonates after bathing with or without liquid cleanser on skin barrier function during the first 8 weeks of life [19]. After 8 weeks, median TEWL was significantly lower on the front, abdomen, and upper leg of neonates who received an emollient after taking a bath with liquid cleanser (*P *< .001 for all regions versus infants who bathed with water alone and did not receive an emollient after bathing). After 8 weeks, median TEWL also was significantly lower on the forehead, abdomen, upper leg, and buttock in neonates who received an emollient after bathing with water alone (*P *< .001 for all listed regions versus infants who bathed with water alone and did not receive an emollient). Emollient use after bathing with or without a liquid cleanser led to an improvement in SC hydration on the forehead and abdomen (*P *< .001 versus infants who bathed with water alone and did not receive an emollient). Moreover, use of an emollient did not affect skin surface pH or sebum production.

Many healthcare practitioners and caregivers understand the utility of incorporating mild, appropriately formulated cleansers into the bathing routine, yet far fewer caregivers recognize the importance or benefits of emollient use for application on healthy neonatal and infant skin. In a recent study, 90% of the mothers surveyed believed that their child's skin was not dry, yet clinical evaluation revealed that only 37% of these children had nondry skin, whereas the remaining children exhibited clinical signs of low to moderately dry skin [102].

### 7.1. Formulation Considerations

Similar to the case of cleansing products, appropriate formulation of emollient products need to take into account the particular nature of infant skin properties [7, 11]. Some considerations that may be important when selecting a skin care emollient product are summarized in Table 4. Although this table is not meant to be an exhaustive list, we have attempted to provide practical considerations relating to preservative systems, fragrances, and the reasons behind other formulation considerations.

It has been postulated that emollient products containing a physiologic balance of epidermal lipids (3 : 1 : 1 : 1 molar ratio of cholesterol/ceramide/palmitate/linoleate) are optimal for barrier repair [103]. Furthermore, many compounds (used alone or in combination with other molecules) have been reported to have beneficial effects on skin barrier function. However, due to the complex nature of emollient formulations and differing individual needs, designing emollients that are optimized for a particular individual and tailoring the emollient for maximum efficacy are still active areas of research [104].

Oils are used traditionally in some countries as emollients during the bathing process [105–109], to treat hypothermia in newborns, [110], or to remove impurities from neonatal skin hours after birth [111]. Some dermatologists have recommended using bath oils for their ability to leave a film on the skin surface or to reduce xerosis [106–108]. One study [109] found that bath oils can be beneficial to infants, yet another double-blind, randomized study showed that some bath or shower oils can be irritating to skin [112]. More recently, an analysis of systematic review found that there was no benefit associated with using oils to treat conditions like atopic eczema [113]. As noted by Shams et al. [113], there is an absence of evidence demonstrating a benefit of using bath emollients in addition to directly applied emollients in the treatment of atopic eczema. Furthermore, Tarr and Iheanacho [114] were not able to find a randomized controlled trial that showed the benefit of using bath emollients. Although the benefits of using oils to improve the skin barrier remain equivocal, bath oil use may have a soothing or calming effect on infants when used during massage or bathing [115, 116]. Moreover, the incorporation of emollients into the bathing routine may provide emotional benefits such as reinforcement of the parental or caregiver bond through touch [29].

While bath oils may not have an obvious benefit, some emollient formulations contain essential fatty acids (e.g., linoleic acid) that can provide systemic benefits to neonates [117]. Not all vegetable oils are appropriate for use on skin [118]. Vegetable oils can vary in composition, for example, in the ratio of linoleic to oleic acid. Some vegetable oils, including certain olive, soybean, and mustard oils, can be detrimental to the integrity of the skin barrier [119]. Some unsaturated free fatty acids can act as permeation enhancers [120], an effect that may cause contact dermatitis in adults [121–124]. In addition, many vegetable oils are unstable and degrade by hydrolysis and oxidation. Degradation can increase the likelihood of microbial growth and spoilage, especially in hot, humid environments. Cutaneous *Propionibacterium acnes* and *Propionibacterium granulosum* secrete lipases, enzymes that hydrolyze sebum triglycerides to free fatty acids [125]. By extension, *Propionibacterium acnes*, *Propionibacterium granulosum*, and possibly other cutaneous bacteria may hydrolyze vegetable oils present in topicals into free fatty acids, accelerating the degradation of vegetable oils on the skin surface. Use of unstable emollients or those that degrade quickly may lead to undesirable effects, especially on infant skin that is undergoing SC maturation and expansion of innate immune function.

Emollients that contain inert, stable ingredients such as mineral oil are preferable for use on the maturing infant skin. Mineral oil, a semiocclusive ingredient that penetrates the upper layers of the SC [126], is immiscible with water. It is noncomedogenic [127], has a long record of safe use [128], and is unlikely to go rancid even in hot, humid climates. Mineral oil helps to enhance the skin barrier as shown by a reduction in TEWL following topical application of the oil [126]. By reducing the amount of evaporated water, it helps keep the SC more hydrated, leading to an improved appearance on the skin surface. Other favorable physical properties of mineral oil include a low viscosity and a low specific gravity relative to water.

**Figure 2:**
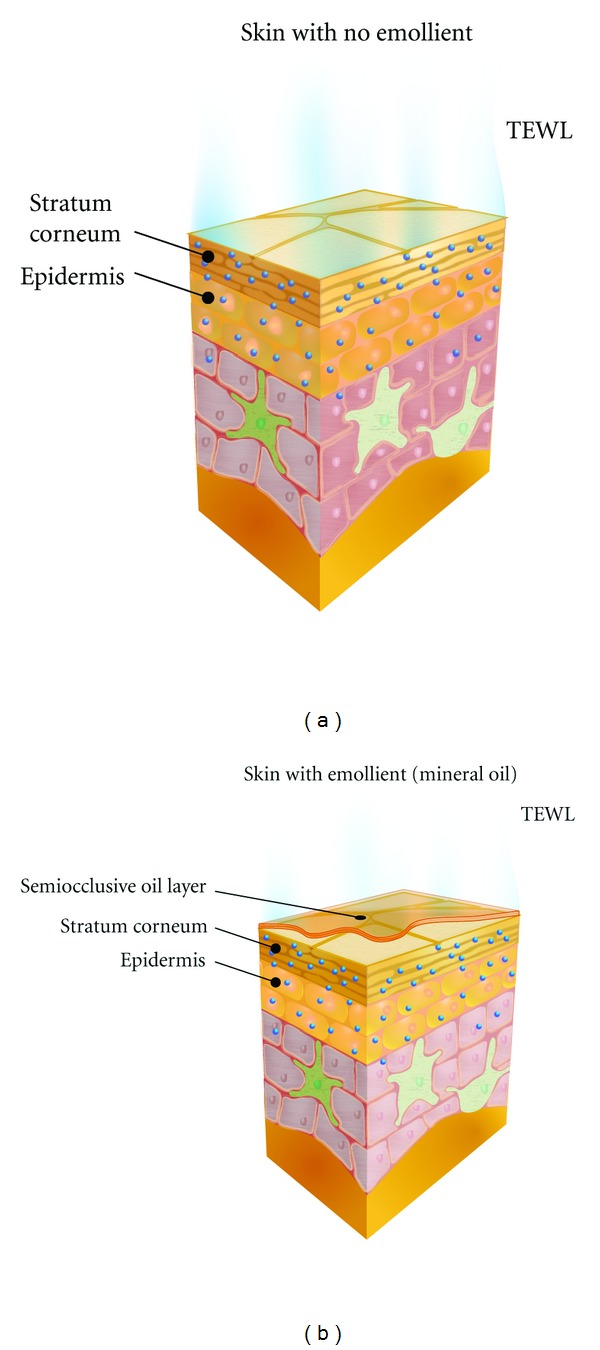
Stratum corneum (SC) moisture retention following application of mineral oil emollient. In (a), transepidermal water loss (TEWL) from the SC is shown under ambient temperature, humidity, and pressure. In (b), TEWL is reduced following emollient application. Oils in the emollient create a semiocclusive layer. The reduction in water evaporation leads to greater water retention in the SC.

 The semiocclusive mineral oil layer on the skin surface helps to retain water by retarding water evaporation [126]. In an unpublished experiment, our group investigated the effects of mineral oil on water retention in excised human SC. Equal weights and sizes of human SC were dehydrated at a constant temperature and humidity for 48 hours. After dehydration, the weights of the human SC samples were recorded. One set of samples (group 1) underwent full hydration by placing the samples in a closed chamber (90% humidity) for 48 hours. At the end of this period, the “wet” sample weight was recorded. A second set of samples (group 2) was allowed to equilibrate to room temperature. Once complete, sample weights in group 2 were recorded. The weight of the hydrated samples was calculated by taking the average percentage of the wet sample weight (group 1) minus the average percentage of the room equilibrated sample weight (group 2). A third set of samples (group 3; control) was maintained at dry weight until use. Mineral oil was applied to the fully hydrated samples in group 1, while two other moisturizing lotions were applied to the samples in group 2. Mineral oil and test lotions were weight-adjusted to ensure that equivalent weights of oil, lotion, and water were applied to human SC (some of the lotions contained water, whereas mineral oil contained none). Weight measurements were taken immediately after product application on all samples; weights also were recorded periodically until there was no further decline in sample weight (i.e., complete evaporation of SC water). In the absence of mineral oil, SC moisture evaporated quickly, whereas samples with mineral oil showed higher water retention.  Figure 2 shows a hypothetical model for how a semiocclusive layer of mineral oil could improve the water barrier. In the left panel, no mineral oil is present. In the right panel, water evaporation from the surface of skin slows in the presence of mineral oil, leading to reduced TEWL.

Another approach to enhance the skin barrier of infant skin is to combine the emollient ingredients within the liquid cleanser formulation [29]. More studies are needed to determine specifically which types of emollient formulations will be optimal for neonatal and infant skin.

## 8. Use of Emollients on Compromised Skin

### 8.1. Premature Infants

Gestational age is strongly linked to epidermal barrier function. The skin barrier of premature infants is injured easily and can serve as a portal of entry for agents, causing serious bacterial infections [13, 129]. Several groups have investigated using vegetable seed oils to improve skin barrier function in premature infants of various ages [28, 100, 119, 130]. Although several studies have shown that emollient use can decrease the frequency of dermatitis or improve skin integrity in very premature newborns [16, 17, 26, 131], there is controversy about the use and effectiveness of emollients in high-risk neonates and infants. 

In 2004, Conner et al. [132] reviewed the effectiveness of prophylactic application of topical ointments on nosocomial sepsis rates and other complications in premature births. In their meta-analysis, they included infants (*n* = 1304) with a gestational age <37 weeks who received an emollient within 96 hours of birth. They found that prophylactic application of topical ointments increased the risk of coagulase negative staphylococcal infection (typical relative risk (RR) 1.31, 95% confidence interval (CI) 1.02–1.70; typical risk difference 0.04, 95% CI 0.00–0.08), any bacterial infection (typical RR 1.19, 95% CI 0.97–1.46; typical risk difference 0.04, 95% CI 0.01–0.08) and nosocomial infection (typical RR 1.20, 95% CI 1.00–1.43; typical risk difference 0.05, 95% CI 0.00–0.09). One limitation of this paper was that it included only four studies [16, 17, 26, 131], which reflects the limited number of studies that had been published at that time. It remains to be seen if the conclusions of the meta-analysis would be applicable for other topicals or emollients. 

In the studies that observed higher rates of infection [16, 26, 131], several possible explanations have been proffered as to the cause.Conner et al. [132] speculate that contamination may have occurred during application of the preservative-free petrolatum ointment or that its use may lead to conditions suitable to proliferation of bacterial organisms.Visscher [133] posits that skin occlusion on extremely low birth weight neonates may have delayed barrier maturation. 

It might be further reasonably speculated that increased rates of nosocomial infections could have been due to use of a preservative-free petrolatum-based ointment that was opened and exposed to pathogenic organisms. Although it is unlikely that a preservative-free petrolatum-based product manufactured using good manufacturing practices would become contaminated, inadvertent addition of excessive moisture from a damp environment (i.e., bathroom) could lead to product contamination. Similar to petrolatum, mineral oil is anhydrous, yet there is evidence that it can become contaminated by improper handling [134]. Given these considerations, formulators should select an effective preservative system, even when formulating low water activity emollients.

Several studies have found very high concentrations (>10^4^ colony-forming units (CFU)/g) of microbial contaminants in consumer products that are poorly preserved or preservative-free [135, 136]. Use of a poorly preserved, contaminated emollient led to an outbreak of *P. aeruginosa* in a neonatal intensive care unit [137]. Furthermore, use of preservative-free white petrolatum has been linked to systemic candidiasis [138].

Since publication of the meta-analysis in 2004 [132], other studies have also investigated emollient use in premature infants or neonates. In a randomized controlled trial, Darmstadt et al. evaluated the efficacy of a petrolatum-based emollient and a sunflower seed oil with high-linoleate content on neonatal mortality rates among hospitalized preterm infants (≤33 weeks gestation) at a large tertiary hospital in Bangladesh [28]. Massaging high-risk infants with the petrolatum-based emollient or the high-linoleate sunflower seed oil led to a reduction in nosocomial bloodstream infections (reduction rates for the respective treatments were 71% (95% CI: 17%–82%) and 41% (95% CI: 4%–63%) relative to no treatment). Moreover, massage with either product led to a significant decrease in neonatal mortality (32% and 26% for the petrolatum-based emollient and the high-linoleate sunflower seed oil, resp.) relative to the standard of care for premature neonates (no treatment). In contrast, use of the same petrolatum-based emollient on extremely premature infants (birth weight 501 to 1000 g) in the United States (and other countries) did not have an effect on neonatal mortality [131]. Darmstadt et al. [28] proposed that differences in trial design, study population, treatment (i.e., access to life-saving interventions), and environmental factors could help explain the differences in neonatal mortality rates observed between the two studies [28, 131].

LeFevre et al. [139] used a Monte Carlo simulation on the data generated by Darmstadt et al. [28] and found that use of the petrolatum-based emollient or sunflower seed oil with high-linoleate content was a cost-effective strategy to improve clinical outcomes. Relative to untreated premature infants, the petrolatum-based emollient and sunflower seed oil had respective costs of US$162 and US$61 per death averted and respective costs of US$5.74 and US$2.15 per year of life lost averted [139]. Although both products were cost-effective strategies to reduce neonatal mortality in a hospital setting, it is not known whether a reduction in mortality also would be observed in a low-resource community setting outside the hospital, which is more typical of a normal birthing environment in Bangladesh and other developing countries [140].

Brandon et al. compared the effects of a composition containing water, polymers, and odorless, nonalcoholic evaporating agents or a petrolatum-based emollient on skin barrier integrity over the first two weeks of life in premature (<33 weeks gestation) infants [141]. A nine-point neonatal skin condition score (NSCS) was used to assess skin dryness, erythema, and skin breakdown. TEWL declined significantly over time; there were no differences in TEWL between treatment groups. The neonatal skin condition scores for infants receiving the petrolatum-based emollient were statistically better than those for infants receiving the aqueous polymeric composition, yet both scores were within normal range. Few infants in either treatment group had skin breakdown.

Although many studies have investigated the use of emollients in children or adults with eczema or AD, very few studies have investigated the use of emollients in healthy, premature, or full-term neonates. A summary of studies that have investigated the use of emollients in healthy, preterm or full-term neonates (0–4 weeks old) or infants (1–6 months old) is shown in Table 5.

## 9. Emollient Use May Lead to Long-Term Improvement in Skin Condition

To our knowledge, there are no randomized controlled trials that have investigated the long-term use of emollients on skin barrier function or overall skin condition. Nevertheless, prophylactic use of emollients that are appropriately formulated for use after birth may produce measurable benefits later in life. To test this hypothesis, some members of our team conducted a 6-week study on 51 infants (aged 3 to 12 months) that consisted of giving the infant participants twice-daily baths with a mild baby cleanser, followed by twice-daily application of one of three marketed lotions (unpublished data). Infants were randomized to receive one of three oil-in-water emollient formulations, each of which contained different types of surfactants and other ingredients. Skin barrier function was assessed indirectly by measuring TEWL and SC hydration (skin conductance) on the upper volar arm and lower dorsal arm. The effect of each lotion varied among the three groups. Results indicated that skin barrier function and SC hydration improved with daily use of only one of the emollients over a period of six weeks. These results suggest that emollient efficacy is related to the specific chemistry and ingredients of the formulation. Although no studies have investigated the long-term use of emollients on infants, long-term emollient use could improve the epidermal skin barrier and improve overall skin condition relative to untreated skin.

## 10. Summary

Although the need for and benefits of good skin hygiene are clear, recommendations for best cleansing and bathing practices remain debated during infancy and early childhood. As infant skin continues to change throughout the first years of life, its dynamic properties need to be addressed with appropriate skin care routines. Use of mild surfactant systems in cleansers can enable maintenance of skin barrier integrity; these cleansers may also be minimally disruptive to skin surface pH and have minimal potential to stimulate the production of IL-1*α* and other proinflammatory molecules. Emollients can provide benefits to premature infants or infants with compromised skin barrier function. Few studies to date have demonstrated the benefits of emollient use on healthy, full-term infants. In addition to providing short-term benefits such as maintaining or improving skin barrier function, it is hypothesized that long-term use of emollients may produce lasting benefits to skin barrier function and overall skin condition. In the future, harmonization of neonatal and infant skin care guidelines—including use of properly formulated cleansers and emollients—is warranted.

## Figures and Tables

**Table 1:** Infant and adult skin: similarities and differences.

Structural differences	Infant skin	Adult skin	Reference
Epidermis			
Corneocytes	Smaller	Larger	[2]
Granular cells	Smaller	Larger	[2]
Stratum corneum and epidermis	Thinner	Thicker	[1, 2]
Microrelief lines	More dense	Less dense	[2]
Depth of surface glyphics	Similar to adult	—	[2]
Facultative pigmentation (melanin)	Less	More	[142, 143]
Dermis			
Dermal papillae (density, size, and morphology)	More homogeneous	Less homogeneous	[2]
Distinct papillary-to-reticular dermis transition	Absent	Present	[2]

Compositional differences			

Epidermis			
Natural moisturizing factor concentration	Lower	Higher	[11]
pH	Higher (newborn only)	Lower	[6, 32, 34]
Sebum	Lower (7–12 month-old infant)	Higher	[144]
Stratum corneum water content	Higher	Lower	[11]
Dermis			
Collagen fiber density	Lower	Higher (young adult)	[2, 145]

Functional differences			

Rate of water absorption	Higher	Lower	[11]
Rate of water desorption	Higher	Lower	[11]
Skin barrier function	Competent	Competent	[9, 10]
Transepidermal water loss	Higher	Lower	[11]

**Table 2:** Ideal properties of appropriately formulated cleansers for neonates and infants.

Property	Traditional cleanser	Infant cleanser
Surfactant systems	Amphoteric, anionic	Amphoteric, nonionic, and ethoxylated anionic
Micelle diameter	Smaller	Larger
pH	Slightly acidic to neutral pH	pH should cause minimal changes to skin surface pH
Estimated IL-1ra/IL-1*α* ratio	Larger	Smaller
Preservative system	Some claim preservative-free	Product should be “microbiologically robust”
Fragrance (parfum/perfume)	Higher concentration level	Lower concentration level; restrictions on specific fragrance components; fragranced product clinically evaluated for irritation and sensitization potential
Other	—	Product should be efficacious and should be demonstrated to be well tolerated

IL-1*α*: interleukin-1*α*, IL-1ra: interleukin-1 receptor antagonist.

**Table 3:** Proinflammatory activity of commercially available cleansing products.

Cleanser	IL-1*α* (pg/mL)	MTT cell proliferation assay
Mild baby cleanser	100.5 ± 35.0	99.5%
Sensitive skin syndet bar	1150.1 ± 0.1	6.5%

Mean (± standard deviation) IL-1*α* (pg/mL) released from *in vitro* skin tissue equivalents (EpiDerm, MatTek Corporation, Ashland, MA, USA) after exposure to various cleansing products. MTT cell proliferation (cell viability) assay data are also shown. The sensitive skin syndet bar had significantly more cell death than the mild baby cleanser, IL-1*α*: interleukin-1*α*.

**Table 4:** Practical considerations for emollient product selection.

Efficacy considerations
(i) Appropriate tests should testify to the efficacy of the product formulation

Safety considerations: overall

(i) The margin of safety for each ingredient at the concentration used in the formulation should be considered
(ii) Ingredients in a product can behave differently than in isolation; therefore, it is important to evaluate the full formulation for safety and
potential dermal effects, including irritation and sensitization

Safety considerations: fragrance

(i) The use of fragranced products for healthy neonates and infants should be supported by evidence for safety and tolerance
(ii) Fragrances should be compliant with the International Fragrance Association (IFRA), which is a body that helps to ensure the safety of
fragrance materials

Safety considerations: preservatives

(i) Products should be microbiologically robust
(ii) “Natural” does not always mean safer (e.g., some natural oils (eucalyptus, sage, and tea tree oils) can be toxic at certain levels)
(iii) Preservative ingredients can be natural or synthetic as long as their safety profile is documented; identical chemical structure means
identical safety profile

Safety considerations: labeling and packaging

(i) Directions for product use should communicate and educate parents on safe and appropriate use
(ii) Package design should help to minimize product contamination (e.g., loose top or seal could expose product to microbes)

**Table 5:** Emollient therapy in healthy, full-term, or premature neonates (0–4 weeks old) or infants (1–6 months old) on skin barrier function: literature review^†^.

Healthy, full-term infants					
Study	Cohort	Treatment	Study duration	Endpoints/ measurements	Primary outcome(s)
Garcia Bartels et al. [19]	64 healthy, full-term neonates (gestation ≥37 weeks aged ≤48 hours)	Body wash; body wash with emollient use after bathing; water alone, followed by emollient after bathing	8 weeks	TEWL, SC hydration, skin surface pH, sebum, NSCS, and bacterial colonization	Wash with emollient improved skin condition; in some cases, lower TEWL and higher SC hydration were observed; no adverse events
Garcia Bartels et al. [146]	44 healthy, full-term infants (≥37 weeks gestation) aged 3–6 months old	Lotion was applied after a swimming lesson once weekly or no treatment	5 weeks	TEWL, SC hydration, skin surface pH, and sebum	Reduced TEWL in both groups; site-specific differences in the treatment group were observed
Lowe et al. [147]	10 healthy, full-term neonates (0–4 weeks old; gestation ≥36 weeks) with a family history of allergic disease	Emollient consisting of ceramides, cholesterol, and free fatty acids at a 3 : 1 : 1 ratio and 2% petrolatum (applied once daily)	6 weeks	TEWL, SC hydration, skin surface pH, and sebum	Emollient use reduced TEWL
Simpson et al. [20]	22 full-term infants (≥37 weeks gestation) considered to be at high risk for developing atopic dermatitis	Oil-in-water, petrolatum-based emollient cream	Up to 2 years	TEWL and skin capacitance	Skin barrier measurements remained within normal range; only three participants developed atopic dermatitis
Premature Infants					
Beeram et al. [18]	54 infants (≤27 weeks gestation)	Petrolatum-based emollient applied every 6 hours or no treatment	2 weeks	Fluids, electrolytes, bilirubin, and sepsis	The petrolatum-based emollient led to a significant reduction in the need for fluids; it also led to better urine output, more stable electrolytes, and lower bilirubin values
Brandon et al. [141]	69 infants (<33 weeks gestation)	Polymer, liquid-based film (applied twice) or petrolatum-based emollient (twice-daily application)	2 weeks	Total fluid intake, TEWL, and neonatal skin condition	Both treatments were well tolerated; both led to a decrease in TEWL
Darmstadt et al. [28, 100, 148]	497 premature infants (≤72 hours old; gestation ≤33 weeks)	Sunflower seed oil or petrolatum-based emollient (3 times daily for 14 days, then twice daily until hospital discharge) or no treatment	≥14 days	Survival rate and rate of nosocomial infection	Sunflower seed oil and petrolatum-based emollient reduced mortality by 25–30%; sunflower seed oil reduced nosocomial infection rates by a statistically significant margin
Lane and Drost [16]	34 neonates (29–36 weeks gestation)	Twice-daily application of a water-in-oil emollient; no treatment	16 days	TEWL, NSCS, and quantitative microbiology	Emollient decreased dermatitis of the hands (days 2–11), feet (days 2–16), and abdomen (days 7–11); no changes in microbial flora
Nopper et al. [17]	60 neonates (<33 weeks gestation)	Petrolatum-based emollient (applied twice daily); no treatment	2 weeks	Temperature, TEWL, fluid intake, weight analysis, skin condition, microbiology, and blood/urine analysis for cerebrospinal fluid cultures	Emollient use led to statistically significant decrease in TEWL; significant improvement in infant skin condition on days 7 and 14; less colonization of the axilla on days 2, 3, 4, and 14; statistically significant reduction of positive findings in blood and cerebrospinal fluid

TEWL: transepidermal water loss, SC: stratum corneum, NSCS: neonatal skin condition score.

^†^Studies published between 1 January 1960 and 1 June 2012 were identified by searching peer-reviewed literature indexed in PubMed. The titles and abstracts of indexed publications listed in PubMed were searched using the following words: “newborn OR neonat* OR infant*” (group 1), “emollient OR lotion OR cream OR topical” (group 2), and “skin” (group 3). These three groupings were connected using the Boolean operators “AND”. The titles and abstracts were also searched using the word “vitro” and the Boolean operator “NOT”. Finally, only the titles of PubMed-indexed publications were searched using a fifth group of words and were connected to the search string using the Boolean operator “NOT”: “injury OR wound OR burn OR damage OR eczema OR dermatitis OR psoriasis OR disease* OR pain OR hemangioma* OR syndrome OR sepsis OR antisepsis.” Review articles, publications that were printed in a language other than English were also excluded. Although our search generated 220 publications, only 9 (summarized in Table 5) met the search criteria described above.

## References

[1] Chiou YB, Blume-Peytavi U (2004). Stratum corneum maturation. A review of neonatal skin function. *Skin Pharmacology and Physiology*.

[2] Stamatas GN, Nikolovski J, Luedtke MA, Kollias N, Wiegand BC (2010). Infant skin microstructure assessed in vivo differs from adult skin in organization and at the cellular level. *Pediatric Dermatology*.

[3] Larson AA, Dinulos JGH (2005). Cutaneous bacterial infections in the newborn. *Current Opinion in Pediatrics*.

[4] Meyer-Hoffert U, Zimmermann A, Czapp M (2011). Flagellin delivery by *Pseudomonas aeruginosa* rhamnolipids induces the antimicrobial protein psoriasin in human skin. *PLoS ONE*.

[5] Elias PM (2007). The skin barrier as an innate immune element. *Seminars in Immunopathology*.

[6] Yosipovitch G, Maayan-Metzger A, Merlob P, Sirota L (2000). Skin barrier properties in different body areas in neonates. *Pediatrics*.

[7] Stamatas GN, Nikolovski J, Mack MC, Kollias N (2011). Infant skin physiology and development during the first years of life: a review of recent findings based on in vivo studies. *International Journal of Cosmetic Science*.

[8] Hoath SB, Maibach HI (2003). *Neonatal Skin: Structure and Function*.

[9] Evans NJ, Rutter N (1986). Development of the epidermis in the newborn. *Biology of the Neonate*.

[10] Fluhr JW, Darlenski R, Taieb A (2010). Functional skin adaptation in infancy—almost complete but not fully competent. *Experimental Dermatology*.

[11] Nikolovski J, Stamatas GN, Kollias N, Wiegand BC (2008). Barrier function and water-holding and transport properties of infant stratum corneum are different from adult and continue to develop through the first year of life. *The Journal of Investigative Dermatology*.

[12] Capone KA, Dowd SE, Stamatas GN, Nikolovski J (2011). Diversity of the human skin microbiome early in life. *The Journal of Investigative Dermatology*.

[13] Kalia YN, Nonato LB, Lund CH, Guy RH (1998). Development of skin barrier function in premature infants. *The Journal of Investigative Dermatology*.

[14] Verdier-Sévrain S, Bonté F (2007). Skin hydration: a review on its molecular mechanisms. *Journal of Cosmetic Dermatology*.

[15] Walker L, Downe S, Gomez L (2005). Skin care in the well term newborn: two systematic reviews. *Birth*.

[16] Lane AT, Drost SS (1993). Effects of repeated application of emollient cream to premature neonates’ skin. *Pediatrics*.

[17] Nopper AJ, Horii KA, Sookdeo-Drost S, Wang TH, Mancini AJ, Lane AT (1996). Topical ointment therapy benefits premature infants. *The Journal of Pediatrics*.

[18] Beeram M, Olvera R, Krauss D, Loughran C, Petty M (2006). Effects of topical emollient therapy on infants at or less than 27 weeks’ gestation. *Journal of the National Medical Association*.

[19] Garcia Bartels N, Scheufele R, Prosch F (2010). Effect of standardized skin care regimens on neonatal skin barrier function in different body areas. *Pediatric Dermatology*.

[20] Simpson EL, Berry TM, Brown PA, Hanifin JM (2010). A pilot study of emollient therapy for the primary prevention of atopic dermatitis. *Journal of the American Academy of Dermatology*.

[21] Walters RM, Fevola MJ, LiBrizzi JJ, Martin K (2008). Designing cleansers for the unique needs of baby skin. *Cosmetics & Toiletries*.

[22] McNally NJ, Williams HC, Phillips DR (1998). Atopic eczema and domestic water hardness. *The Lancet*.

[23] Miyake Y, Yokoyama T, Yura A, Iki M, Shimizu T (2004). Ecological association of water hardness with prevalence of childhood atopic dermatitis in a Japanese urban area. *Environmental Research*.

[24] Thomas KS, Dean T, O’Leary C (2011). A randomised controlled trial of ion-exchange water softeners for the treatment of eczema in children. *PLoS Medicine*.

[25] Thomas KS, Koller K, Dean T (2011). A multicentre randomised controlled trial and economic evaluation of ion-exchange water softeners for the treatment of eczema in children: the Softened Water Eczema Trial (SWET). *Health Technology Assessment*.

[26] Pabst RC, Starr KP, Qaiyumi S, Schwalbe RS, Gewolb IH (1999). The effect of application of Aquaphor on skin condition, fluid requirements, and bacterial colonization in very low birth weight infants. *Journal of Perinatology*.

[27] Buraczewska I, Berne B, Lindberg M, Törmä H, Lodén M (2007). Changes in skin barrier function following long-term treatment with moisturizers, a randomized controlled trial. *The British Journal of Dermatology*.

[28] Darmstadt GL, Saha SK, Ahmed ASMNU (2008). Effect of skin barrier therapy on neonatal mortality rates in preterm infants in Bangladesh: a randomized, controlled, clinical trial. *Pediatrics*.

[29] Blume-Peytavi U, Cork MJ, Faergemann J, Szczapa J, Vanaclocha F, Gelmetti C (2009). Bathing and cleansing in newborns from day 1 to first year of life: recommendations from a European round table meeting. *Journal of the European Academy of Dermatology and Venereology*.

[30] Fairley JA, Rasmussen JE (1983). Comparison of stratum corneum thickness in children and adults. *Journal of the American Academy of Dermatology*.

[31] Saijo S, Tagami H (1991). Dry skin of newborn infants: functional analysis of the stratum corneum. *Pediatric Dermatology*.

[32] Hoeger PH, Enzmann CC (2002). Skin physiology of the neonate and young infant: a prospective study of functional skin parameters during early infancy. *Pediatric Dermatology*.

[33] Visscher MO, Chatterjee R, Munson KA, Pickens WL, Hoath SB (2000). Changes in diapered and nondiapered infant skin over the first month of life. *Pediatric Dermatology*.

[34] Giusti F, Martella A, Bertoni L, Seidenari S (2001). Skin barrier, hydration, and pH of the skin of infants under 2 years of age. *Pediatric Dermatology*.

[35] Wanke I, Steffen H, Christ C (2011). Skin commensals amplify the innate immune response to pathogens by activation of distinct signaling pathways. *The Journal of Investigative Dermatology*.

[36] Hanley K, Jiang Y, Elias PM, Feingold KR, Williams ML (1997). Acceleration of barrier ontogenesis *in vitro* through air exposure. *Pediatric Research*.

[37] Rawlings AV (2003). Trends in stratum corneum research and the management of dry skin conditions. *International Journal of Cosmetic Science*.

[38] Rawlings AV, Scott LR, Harding CR, Bowser PA (1994). Stratum corneum moisturization at the molecular level. *The Journal of Investigative Dermatology*.

[39] Machado M, Salgado TM, Hadgraft J, Lane ME (2010). The relationship between transepidermal water loss and skin permeability. *International Journal of Pharmaceutics*.

[40] Rawlings A, Harding C, Watkinson A, Banks J, Ackerman C, Sabin R (1995). The effect of glycerol and humidity on desmosome degradation in stratum corneum. *Archives of Dermatological Research*.

[41] Tagami H, Kanamaru Y, Inoue K (1982). Water sorption-desorption test of the skin in vivo for functional assessment of the stratum corneum. *The Journal of Investigative Dermatology*.

[42] Bouwstra JA, de Graaff A, Gooris GS, Nijsse J, Wiechers JW, van Aelst AC (2003). Water distribution and related morphology in human stratum corneum at different hydration levels. *The Journal of Investigative Dermatology*.

[43] Sato J, Yanai M, Hirao T, Denda M (2000). Water content and thickness of the stratum corneum contribute to skin surface morphology. *Archives of Dermatological Research*.

[44] Pierard GE, Goffin V, Hermanns-Le T, Pierard-Franchimont C (2000). Corneocyte desquamation. *International Journal of Molecular Medicine*.

[45] Engelke M, Jensen JM, Ekanayake-Mudiyanselage S, Proksch E (1997). Effects of xerosis and ageing on epidermal proliferation and differentiation. *The British Journal of Dermatology*.

[46] Rogiers V, EEMCO Group (2001). EEMCO guidance for the assessment of transepidermal water loss in cosmetic sciences. *Skin Pharmacology and Applied Skin Physiology*.

[47] Rippke F, Schreiner V, Schwanitz HJ (2002). The acidic milieu of the horny layer: new findings on the physiology and pathophysiology of skin pH. *American Journal of Clinical Dermatology*.

[48] Lund C, Kuller J, Lane A, Lott JW, Raines DA (1999). Neonatal skin care: the scientific basis for practice. *Neonatal Network*.

[49] Lambers H, Piessens S, Bloem A, Pronk H, Finkel P (2006). Natural skin surface pH is on average below 5, which is beneficial for its resident flora. *International Journal of Cosmetic Science*.

[50] Askin DF (1995). Bacterial and fungal infections in the neonate. *Journal of Obstetric, Gynecologic, and Neonatal Nursing*.

[51] Larsen FSchultz, Diepgen T, Svensson A (1996). The occurrence of atopic dermatitis in North Europe: an international questionnaire study. *Journal of the American Academy of Dermatology*.

[52] Laughter D, Istvan JA, Tofte SJ, Hanifin JM (2000). The prevalence of atopic dermatitis in Oregon schoolchildren. *Journal of the American Academy of Dermatology*.

[53] Werner YLVA, Lindberg M (1985). Transepidermal water loss in dry and clinically normal skin in patients with atopic dermatitis. *Acta Dermato-Venereologica*.

[54] Seidenari S, Giusti G (1995). Objective assessment of the skin of children affected by atopic dermatitis: a study of pH, capacitance and TEWL in eczematous and clinically uninvolved skin. *Acta Dermato-Venereologica*.

[55] Ogawa H, Yoshiike T (1992). Atopic dermatitis: studies of skin permeability and effectiveness of topical PUVA treatment. *Pediatric Dermatology*.

[56] Kong HH, Oh J, Deming C (2012). Temporal shifts in the skin microbiome associated with disease flares and treatment in children with atopic dermatitis. *Genome Research*.

[57] Schittek B (2011). The antimicrobial skin barrier in patients with atopic dermatitis. *Current Problems in Dermatology*.

[58] Lebwohl M, Herrmann LG (2005). Impaired skin barrier function in dermatologic disease and repair with moisturization. *Cutis; Cutaneous Medicine for the Practitioner*.

[59] Hanifin JM, Cooper KD, Ho VC (2004). Guidelines of care for atopic dermatitis, developed in accordance with the American Academy of Dermatology (AAD)/American Academy of Dermatology Association ‘Administrative Regulations for Evidence-Based Clinical Practice Guidelines’. *Journal of the American Academy of Dermatology*.

[60] National Collaborating Centre for Women's and Children's Health NICE clinical guideline 57. Atopic eczema in children. http://www.nice.org.uk/nicemedia/live/11901/38597/38597.pdf.

[61] Hon KLE, Wong KY, Cheung LK (2007). Efficacy and problems associated with using a wet-wrap garment for children with severe atopic dermatitis. *The Journal of Dermatological Treatment*.

[62] Cox H, Lloyd K, Williams H (2011). Emollients, education and quality of life: the RCPCH care pathway for children with eczema. *Archives of Disease in Childhood*.

[63] Stamatas GN, Zerweck C, Grove G, Martin KM (2011). Documentation of impaired epidermal barrier in mild and moderate diaper dermatitis in vivo using noninvasive methods. *Pediatric Dermatology*.

[64] Adalat S, Wall D, Goodyear H (2007). Diaper dermatitis-frequency and contributory factors in hospital attending children. *Pediatric Dermatology*.

[65] Atherton DJ (2001). The aetiology and management of irritant diaper dermatitis. *Journal of the European Academy of Dermatology and Venereology*.

[66] Visscher MO, Chatterjee R, Munson KA, Bare DE, Hoath SB (2000). Development of diaper rash in the newborn. *Pediatric Dermatology*.

[67] Atherton DJ (2004). A review of the pathophysiology, prevention and treatment of irritant diaper dermatitis. *Current Medical Research and Opinion*.

[68] Gupta AK, Skinner AR (2004). Management of diaper dermatitis. *International Journal of Dermatology*.

[69] Scheinfeld N (2005). Diaper dermatitis: a review and brief survey of eruptions of the diaper area. *American Journal of Clinical Dermatology*.

[70] Shin HT (2005). Diaper dermatitis that does not quit. *Dermatologic Therapy*.

[71] Humphrey S, Bergman JN, Au S (2006). Practical management strategies for diaper dermatitis. *Skin Therapy Letter*.

[72] (1974). American Academy of Pediatrics Committee on Fetus and Newborn. Skin care of newborns. *Pediatrics*.

[73] Bergström A, Byaruhanga R, Okong P (2005). The impact of newborn bathing on the prevalence of neonatal hypothermia in Uganda: a randomized, controlled trial. *Acta Paediatrica, International Journal of Paediatrics*.

[74] Lund C, Kuller J, Raines D, Ecklund S, Archambault M, O’Flaherty P (2007). *Neonatal Skin Care*.

[75] National Collaborating Centre for Primary Care NICE clinical guideline 37. Routine postnatal care of women and their babies. http://www.nice.org.uk/nicemedia/live/10988/30144/30144.pdf.

[76] Gelmetti C (2001). Skin cleansing in children. *Journal of the European Academy of Dermatology and Venereology*.

[77] Kuehl BL, Fyfe KS, Shear NH (2003). Cutaneous cleansers. *Skin Therapy Letter*.

[78] Afsar FS (2009). Skin care for preterm and term neonates. *Clinical and Experimental Dermatology*.

[79] Gfatter R, Hackl P, Braun F (1997). Effects of soap and detergents on skin surface pH, stratum corneum hydration and fat content in infants. *Dermatology*.

[80] Darmstadt GL, Dinulos JG (2000). Neonatal skin care. *Pediatric Clinics of North America*.

[81] Ananthapadmanabhan KP, Moore DJ, Subramanyan K, Misra M, Meyer F (2004). Cleansing without compromise: the impact of cleansers on the skin barrier and the technology of mild cleansing. *Dermatologic Therapy*.

[82] Siegfried EC, Shah PY (1999). Skin care practices in the neonatal nursery: a clinical survey. *Journal of Perinatology*.

[83] Dizon MV, Galzote C, Estanislao R, Mathew N, Sarkar R (2010). Tolerance of baby cleansers in infants: a randomized controlled trial. *Indian Pediatrics*.

[84] Löffler H, Happle R (2003). Profile of irritant patch testing with detergents: sodium lauryl sulfate, sodium laureth sulfate and alkyl polyglucoside. *Contact Dermatitis*.

[85] Charbonnier V, Morrison BM, Paye M, Maibach HI (2001). Subclinical, non-erythematous irritation with an open assay model (washing): sodium lauryl sulfate (SLS) versus sodium laureth sulfate (SLES). *Food and Chemical Toxicology*.

[86] Robinson VC, Bergfeld WF, Belsito DV (2010). Final report of the amended safety assessment of sodium laureth sulfate and related salts of sulfated ethoxylated alcohols. *International Journal of Toxicology*.

[87] Subramanyan K (2004). Role of mild cleansing in the management of patient skin. *Dermatologic Therapy*.

[88] Fevola MJ, Walters RM, LiBrizzi JJ (2010). A new approach to formulating mild cleansers: hydrophobically-modified polymers for irritation mitigation. *Polymeric Delivery of Therapeutics*.

[89] White MI, Jenkinson DM, Lloyd DH (1987). The effect of washing on the thickness of the stratum corneum in normal and atopic individuals. *The British Journal of Dermatology*.

[90] Parra JL, Paye M, EEMCO Group (2003). EEMCO guidance for the in vivo assessment of skin surface pH. *Skin Pharmacology and Applied Skin Physiology*.

[91] Chollopetz da Cunha ML, Procianoy RS (2005). Effect of bathing on skin flora of preterm newborns. *Journal of Perinatology*.

[92] Gallo RL, Nakatsuji T, EEMCO Group (2011). Microbial symbiosis with the innate immune defense system of the skin. *The Journal of Investigative Dermatology*.

[93] Bernhofer LP, Barkovic S, Appa Y, Martin KM (1999). IL-1*α* and IL-1ra secretion from epidermal equivalents and the prediction of the irritation potential of mild soap and surfactant-based consumer products. *Toxicology In Vitro*.

[94] Bernhofer LP, Seiberg M, Martin KM (1999). The influence of the response of skin equivalent systems to topically applied consumer products by epithelial-mesenchymal interactions. *Toxicology In Vitro*.

[95] Hirao T, Aoki H, Yoshida T, Sato Y, Kamoda H (1996). Elevation of interleukin 1 receptor antagonist in the stratum corneum of sun-exposed and ultraviolet B-irradiated human skin. *The Journal of Investigative Dermatology*.

[96] Perkins MA, Osterhues MA, Farage MA, Robinson MK (2001). A noninvasive method to assess skin irritation and compromised skin conditions using simple tape adsorption of molecular markers of inflammation. *Skin Research and Technology*.

[97] Terui T, Hirao T, Sato Y (1998). An increased ratio of interleukin-1 receptor antagonist to interleukin-1*α* in inflammatory skin diseases. *Experimental Dermatology*.

[98] Goffin V, Paye M, Piérard GE (1995). Comparison of *in vitro* predictive tests for irritation induced by anionic surfactants. *Contact Dermatitis*.

[99] Eichenfield LF, Hardaway CA (1999). Neonatal dermatology. *Current Opinion in Pediatrics*.

[100] Darmstadt GL, Saha SK, Ahmed ASMNU (2005). Effect of topical treatment with skin barrier-enhancing emollients on nosocomial infections in preterm infants in Bangladesh: a randomised controlled trial. *The Lancet*.

[101] Schürer N, Schliep V, Williams ML (1995). Differential utilization of linoleic and arachidonic acid by cultured human keratinocytes. *Skin Pharmacology*.

[102] Tierney NK, Stamatas GN, Lodén  M, Maibach HI (2012). Update on infant skin with special focus on dryness and the impact of moisturizers. *Treatment of Dry Skin Syndrome*.

[103] Bhutta ZA, Darmstadt GL, Hasan BS, Haws RA (2005). Community-based interventions for improving perinatal and neonatal health outcomes in developing countries: a review of the evidence. *Pediatrics*.

[104] Lodén M (2003). Do moisturizers work?. *Journal of Cosmetic Dermatology*.

[105] James AP (1961). Bath oils in the management of dry, pruritic skin. *Journal of the American Geriatrics Society*.

[106] Stanfield JW, Levy J, Kyriakopoulos AA, Waldman PM (1981). A new technique for evaluating bath oil in the treatment of dry skin. *Cutis; Cutaneous Medicine for the Practitioner*.

[107] Stender IM, Blichmann C, Serup J (1990). Effects of oil and water baths on the hydration state of the epidermis. *Clinical and Experimental Dermatology*.

[108] Hill S, Edwards C (2002). A comparison of the effects of bath additives on the barrier function of skin in normal volunteer subjects. *The Journal of Dermatological Treatment*.

[109] Bettzuege-Pfaff BI, Melzer A (2005). Treating dry skin and pruritus with a bath oil containing soya oil and lauromacrogols. *Current Medical Research and Opinion*.

[110] Ogunlesi TA, Ogunfowora OB, Ogundeyi MM (2009). Prevalence and risk factors for hypothermia on admission in Nigerian babies <72 h of age. *Journal of Perinatal Medicine*.

[111] Iweze FA (1983). Taboos of childbearing and child-rearing in Bendel state of Nigeria. *Journal of Nurse-Midwifery*.

[112] Lodén M, Buraczewska I, Edlund F (2004). Irritation potential of bath and shower oils before and after use: a double-blind randomized study. *The British Journal of Dermatology*.

[113] Shams K, Grindlay DJC, Williams HC (2011). What’s new in atopic eczema? An analysis of systematic reviews published in 2009-2010. *Clinical and Experimental Dermatology*.

[114] Tarr A, Iheanacho I (2009). Should we use bath emollients for atopic eczema?. *British Medical Journal*.

[115] Field T, Schanberg S, Davalos M, Malphurs J (1996). Massage with oil has more positive effects on normal infants. *Pre- and Perinatal Psychology Journal*.

[116] Field T, Field T, Cullen C (2008). Lavender bath oil reduces stress and crying and enhances sleep in very young infants. *Early Human Development*.

[117] Friedman Z, Shochat SJ, Maisels MJ, Marks KH, Lamberth EL (1976). Correction of essential fatty acid deficiency in newborn infants by cutaneous application of sunflower seed oil. *Pediatrics*.

[118] Maghraby GMMEl, Campbell M, Finnin BC (2005). Mechanisms of action of novel skin penetration enhancers: phospholipid versus skin lipid liposomes. *International Journal of Pharmaceutics*.

[119] Darmstadt GL, Mao-Qiang M, Chi E (2002). Impact of topical oils on the skin barrier: possible implications for neonatal health in developing countries. *Acta Paediatrica*.

[120] Williams AC, Barry BW (2004). Penetration enhancers. *Advanced Drug Delivery Reviews*.

[121] Kränke B, Komericki P, Aberer W (1997). Olive oil—contact sensitizer or irritant?. *Contact Dermatitis*.

[122] Isaksson M, Bruze M (1999). Occupational allergic contact dermatitis from olive oil in a masseur. *Journal of the American Academy of Dermatology*.

[123] Wong GAE, King CM (2004). Occupational allergic contact dermatitis from olive oil in pizza making. *Contact Dermatitis*.

[124] Williams JD, Tate BJ (2006). Occupational allergic contact dermatitis from olive oil. *Contact Dermatitis*.

[125] Puhvel SM, Reisner RM, Sakamoto M (1975). Analysis of lipid composition of isolated human sebaceous gland homogenates after incubation with cutaneous bacteria. Thin-layer chromatography. *The Journal of Investigative Dermatology*.

[126] Patzelt A, Lademann J, Richter H (2012). In vivo investigations on the penetration of various oils and their influence on the skinbarrier. *Skin Research and Technology*.

[127] DiNardo JC (2005). Is mineral oil comedogenic?. *Journal of Cosmetic Dermatology*.

[128] Nash JF, Gettings SD, Diembeck W, Chudowski M, Kraus AL (1996). A toxicological review of topical exposure to white mineral oils. *Food and Chemical Toxicology*.

[129] Darmstadt GL, Ahmed ASMNU, Saha SK (2005). Infection control practices reduce nosocomial infection and mortality in preterm infants in Bangladesh. *Journal of Perinatology*.

[130] Ahmed ASMNU, Saha SK, Chowdhury MA (2007). Acceptability of massage with skin barrier-enhancing emollients in young neonates in Bangladesh. *Journal of Health, Population and Nutrition*.

[131] Edwards WH, Conner JM, Soll RF (2004). The effect of prophylactic ointment therapy on nosocomial sepsis rates and skin integrity in infants with birth weights of 501 to 1000 g. *Pediatrics*.

[132] Conner JM, Soll RF, Edwards WH (2004). Topical ointment for preventing infection in preterm infants. *Cochrane Database of Systematic Reviews*.

[133] Visscher MO (2009). Update on the use of topical agents in neonates. *Newborn and Infant Nursing Reviews*.

[134] Schuchat A, Lizano C, Broome CV, Swaminathan B, Kim C, Winn K (1991). Outbreak of neonatal listeriosis associated with mineral oil. *Pediatric Infectious Disease Journal*.

[135] Brannan DK, Dille JC (1990). Type of closure prevents microbial contamination of cosmetics during consumer use. *Applied and Environmental Microbiology*.

[136] Na’was T, Alkofahi A (1994). Microbial contamination and preservative efficacy of topical creams. *Journal of Clinical Pharmacy and Therapeutics*.

[137] Becks VE, Lorenzoni NM (1995). *Pseudomonas aeruginosa* outbreak in a neonatal intensive care unit: a possible link to contaminated hand lotion. *American Journal of Infection Control*.

[138] Campbell JR, Zaccaria E, Baker CJ (2000). Systemic candidiasis in extremely low birth weight infants receiving topical petrolatum ointment for skin care: a case-control study. *Pediatrics*.

[139] LeFevre A, Shillcutt SD, Saha SK (2010). Cost-effectiveness of skin-barrier-enhancing emollients among preterm infants in Bangladesh. *Bulletin of the World Health Organization*.

[140] Bharathi M, Sundaram V, Kumar P (2009). Skin barrier therapy and neonatal mortality in preterm infants. *Pediatrics*.

[141] Brandon DH, Coe K, Hudson-Barr D, Oliver T, Landerman LR (2010). Effectiveness of No-Sting skin protectant and Aquaphor on water loss and skin integrity in premature infants. *Journal of Perinatology*.

[142] Brenner M, Hearing VJ (2008). The protective role of melanin against UV damage in human skin. *Photochemistry and Photobiology*.

[143] Mack MC, Tierney NK, Ruvolo E, Stamatas GN, Martin KM, Kollias N (2010). Development of solar UVR-related pigmentation begins as early as the first summer of life. *The Journal of Investigative Dermatology*.

[144] Agache P, Blanc D, Barrand C, Laurent R (1980). Sebum levels during the first year of life. *The British Journal of Dermatology*.

[145] Vitellaro-Zuccarello L, Cappelletti S, Rossi VDalPozzo, Sari-Gorla M (1994). Stereological analysis of collagen and elastic fibers in the normal human dermis: variability with age, sex, and body region. *The Anatomical Record*.

[146] Garcia Bartels N, Rösler S, Martus P (2011). Effect of baby swimming and baby lotion on the skin barrier of infants aged 3–6 months. *Journal der Deutschen Dermatologischen Gesellschaft*.

[147] Lowe AJ, Tang ML, Dharmage SC (2012). A phase i study of daily treatment with a ceramide-dominant triple lipid mixture commencing in neonates. *BMC Dermatology*.

[148] Darmstadt GL, Saha SK, Ahmed ASMNU (2007). Effect of topical emollient treatment of preterm neonates in Bangladesh on invasion of pathogens into the bloodstream. *Pediatric Research*.

